# Applicability of the Dual-Factor Model of Mental Health in the Mental Health Screening of Chinese College Students

**DOI:** 10.3389/fpsyg.2020.549036

**Published:** 2021-01-18

**Authors:** Rong Xiao, Chao Zhang, Qiaozhen Lai, Yanfei Hou, Xiaoyuan Zhang

**Affiliations:** ^1^Department of Psychology, School of Public Health, Southern Medical University, Guangzhou, China; ^2^School of Foreign Studies, Southern Medical University, Guangzhou, China

**Keywords:** dual-factor model of mental health, mental health, screening, flourishing, well-being, depression

## Abstract

Traditional mental health models focus on psychopathological symptoms. In contrast, a dual-factor model of mental health integrates psychopathology and subjective well-being into a mental health continuum, and it is adjustment and supplement for traditional mental health research paradigm. The present study explores the applicability of a dual-factor model of mental health in mental health screening of Chinese college students. To assess mental health statuses of 2,065 college students, we used Flourishing Scale Chinese Version, Satisfaction With Life Scale, the seven-item Patient Health Questionnaire, the Mental Health Continuum–Short Form, and Purpose in Life Test–Short Form. Results showed that the dual-factor model of mental health has a good fit index. Also, a feasible screening scale was addressed. The results indicate the importance of addressing both subjective well-being and psychopathology in evaluating mental health screening of college students.

## Introduction

Greenspoon and Saklofske’s (2001) dual-factor model (DFM) of mental health integrates psychopathology (PTH) and subjective well-being (SWB) into a mental health continuum, and provides an adjustment and supplement to traditional mental health research paradigms. Based on the DFM, mental health status may be further divided into the following four categories: “positive mental health” (manifesting high levels of SWB and low levels of PTH symptoms), “vulnerable” (low levels of PTH but also low SWB), “symptomatic but content” (average to high levels of SWB and high PTH), and “troubled” (low SWB along with high PTH) (Antaramian et al., 2010). In addition, six categories (flourishing group, moderately mentally health group, languishing group, mental illness and flourishing group, mental illness and moderately mentally healthy group, and mental illness and languishing group) of mental health status were also proposed (Keyes, 2007). Of these classification methods, predictors within the DFM include temperament, personality, self-concept, locus of control, and interpersonal relations. Subsequent investigation has supported the two continua model of mental health and illness, as well as the benefits of “flourishing” to individuals and society. Furthermore, it has been established that mental health status is closely related to bodily well-being, academic achievements, and interpersonal relationships (Antaramian et al., 2010; Eklund et al., 2011; Suldo et al., 2011; Renshaw and Cohen, 2014), and the DFM can predict an individual’s future mental health status and behavior (Suldo et al., 2011; Kelly et al., 2012; Xiong et al., 2017). Applicability of the DFM within Chinese populations has also been shown to be usable and reliable (Dong et al., 2014; Hai et al., 2015; Wang et al., 2016; Xiong et al., 2016).

Regarding the choice of appropriate positive psychology indicators, Seligman (2011) theorized that the theme of positive psychology is happiness, and the standard in evaluating happiness concerns an individual’s level of flourishing. Hence, the ultimate goal of positive psychology might be taken to help everybody to achieve flourishing. It is an individual positive mental health state, and flourishing individuals are often passionate and energetic and lead a positive life in personal and social settings (Keyes, 2003). Flourishing structurally involves considerations such as the meaning of life, interpersonal relationships, devotion and interest, capability, self-acceptance, optimism, and esteem. Individual positive psychology can be manifested through levels of flourishing (Diener et al., 2010).

However, although the DFM has been shown to be effective in evaluating mental health, there is currently no consensus regarding DFM indicators, meaning that different evaluations can be obtained based on the adoption of different DFM indicators. Positive indicators include life satisfaction and positive emotions, whereas negative indicators include depression and negative emotions (Dong et al., 2014; Hai et al., 2015). It has been argued that positive and negative indicators based on the DFM should not be confined to specific indicators. Choice of indicators needs to be compliant with population groups (Wang and Zhang, 2011). Nonetheless, unified indicators are essential for community mental health screening and intervention, as well as when comparing the mental health statuses of different population groups, exercising mental health interventions, and allocating mental health resources. Although concept of “flourishing” has been incorporated into evaluations of SWB and positive mental health, a Flourishing Scale (FS) has yet to be applied in mental health assessments based on the DFM (Diener et al., 2010). The most commonly used depression screening tool, the Patient Health Questionnaire (PHQ), has not yet been utilized as part of the DFM either.

Our study proposes to test the applicability of a flourishing–depression combined assessment model within mental health screening, with depression as the negative mental health indicator and flourishing as the positive mental health indicator, in order that empirical evidence might be established to ensure future accurate evaluations and graded interventions. Based on the nine-item PHQ (PHQ-9), we formulated a simplified version, PHQ-7 (Kroenke et al., 2003, 2009). Regarding Greenspoon and Saklofske’s (2001) categorization by four and Keyes’s (2007) categorization by six, we tested both tools in mental health screening of Chinese college students.

## Materials and Methods

### Participants and Procedures

The survey was carried out using snowball sampling, with experimental assistants sending out the website link of the questionnaire using popular Chinese social media sites. The study’s participants were undergraduates from different districts of China. The voluntary and confidential nature of participants’ involvement in this study was clearly communicated to all students involved. Participants gave their informed consent by a tick of informed consent item at the beginning of the questionnaire. The consent procedure was approved by the Academic Ethics Committee of Southern Medical University.

The survey was carried out from November 2016 to January 2017, and we received responses from 2,387 potential participants. Participants who spent too little time in filling out the questionnaire (i.e.,<6 min), who were suspected not to respond sincerely (i.e., all the answers were the same), or who missed more than 20% of the items were excluded. Finally, 2,065 questionnaires were collected, representing an effective response rate of 86.5%. Of this final sample of questionnaires, 781 were completed by male students and 1,284 by female students. Participants’ ages ranged from 17 to 26 years, and the average age was 20.85 years (±1.30 years).

### Measures

#### The Flourishing Scale Chinese Version

Diener et al. (2010) formulated the FS, which features eight items and encompasses well-established psychometric characteristics applicable in different countries. The scale describes human functioning from a broad and comprehensive perspective, covering (1) competence, (2) engagement, (3) meaning and purpose, (4) optimism, (5) self-acceptance, (6) supportive relationships, (7) well-being of others, and (8) being respected. A 7-point Likert-type scale is used to evaluate the items, with scores ranging between 0 and 56 points and higher scores denoting participants’ more positive mental resources and social function. Tang et al. (2016) developed the Chinese version of the FS, which has demonstrated good reliability and validity with the original scale. In our research, the internal reliability was 0.948.

#### Patient Health Questionnaire

Kroenke et al. (2001) compiled a PHQ based on the *Diagnostic and Statistical Manual of Mental Disorders* (*DSM-IV*). PHQ-9 is the depression screening section of the PHQ and aligns with nine depression symptoms described in *DSM-IV*. Owing to the practicability of PHQ-9, it has been widely used in the clinical setting, as well as in scientific research (Patten and Schopflocher, 2009). A Chinese version of the questionnaire has also been shown to have good reliability and validity (Hu et al., 2014).

Several abridged versions (e.g., PHQ-2 and PHQ-8) have been derived from the application of PHQ-9 (Kroenke et al., 2003, 2009). We formulated PHQ-7, in which items 1, 2, 3, 4, 6, 7, and 9 of the original PHQ-9 were retained, but diet disorder (item 5) and bradykinesia (item 8) were excluded. We deleted item 5 because a number of factors would lead to diet disorder, and item 8 because bradykinesia would puzzle participants. Items 5 and 8 did not manifest enough specificity for depression screening. The remaining seven items are more focused on depression screening. The questionnaire was used to assess whether individuals manifested typical symptoms of depression, such as lack of interest or feeling down. A 3-point Likert-type scale was applied, and the maximum total score was 21 points, with higher scores suggesting a greater likelihood of depression.

A receiver operating characteristic (ROC) curve was drawn, with the Self-Rating Depressive Scale (SDS) as the gold standard (Zung, 1965). The area under the curve (AUC) values of PHQ-7, PHQ-9, PHQ-8, and PHQ-2 were found to be 0.840, 0.839, 0.831, and 0.721, respectively (*p* < 0.001). These results indicate that the efficacy of PHQ-7 is no lower than that of PHQ-9 and higher than that of PHQ-2. With an ROC curve drawn using PHQ-9 as the gold standard and PHQ-7 ≥ 8 as the dividing line, the AUC value was found to be 0.986 (standard deviation = 0.003, *p* < 0.001). Depression disorder sensitivity was 93%, and specificity was 96.4%. The efficacy standard relation of PHQ-7 with PHQ-9 and the SDS were 0.967 and 0.625, respectively, which further substantiates the reliability of PHQ-7. Internal reliability with the PHQ-7 was found to be 0.872. Therefore, we inferred that PHQ-7 demonstrates good reliability and validity and that it could be applied in assessments of depression.

#### Mental Health Continuum–Short Form

The Mental Health Continuum–Short Form (MHC-SF) was formulated by Keyes (2005) and is based on measures of emotional, psychological, and social well-being. The original scale featured 40 items, but was simplified to include 14 items (MHC-SF). The Chinese version of the MHC-SF was translated by Yin and He (2012), and its reliability and validity have been shown to fulfill psychometric requirements. In our research, the internal consistency reliability was found to be 0.963.

#### Satisfaction With Life Scale

The Satisfaction With Life Scale (SWLS) features five items that are rated according to a 7-point Likert-type scale. A higher score represents higher life satisfaction (Diener et al., 1985). Owing to its consistent reliability, the scale has been widely used in intercultural research and has been found to be valid and reliable in Chinese contexts (Kroenke et al., 2001). In our research, the internal consistency reliability was calculated to be 0.908.

#### Purpose in Life Test–Short Form

Schulenberg et al. (2011) selected four items from the Purpose in Life Test (PIL) test and formulated the PIL–Short Form (PIL-SF). The four items encompass (1) the presence of clear life goals, (2) life being meaningful, (3) life goal completion, and (4) the presence of goals/life purpose. A 7-point Likert-type scale is applied, and the total scores range from 4 to 28 points. A higher score represents a more purposeful lifestyle. The Chinese version of the PIL-SF showed good reliability and validity. In our research, the internal consistency reliability was found to be 0.864 (Xiao and Li, 2016).

### Analytical Strategy

IBM’s SPSS and Amos 21 was utilized for the structural equation modeling, and SPSS 19.0 was used for the descriptive analysis and single-factor analysis of variance. SEM was applied in testing validity of DFM of mental health (i.e., flourishing–depression and life satisfaction–depression). Structural validity was testified, and proposed parameters were compared. Descriptive analysis and analysis of variance were applied in presenting distribution of mental health status of college students, as well as their differences in FS Chinese Version, SWLS, PHQ-7, the MHC-SF, and PIL-SF.

## Results

### One-Dimensional Analysis of College Students’ Mental Health

According to previous research, the boundary value for PHQ-7 is 8 points. Scores greater than 8 points are regarded as being positive for depression, and those less than 8 points as negative for depression. In our study, 79.6% of our college student participants did not exhibit depression symptoms, whereas 20.4% did show symptoms of depression. In addition, the average score for the FS is 5.18 (±1.09), and the boundary value for the FS is 5 points. In our study, 69.7% of college students manifested high flourishing levels, and 30.3% showed low levels of flourishing.

### Structural Validity of the Mental Health Flourishing–Depression DFM

**FIGURE 1:**
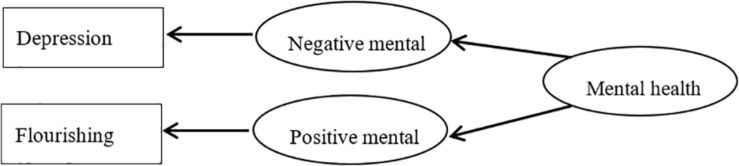
Flourishing-depression of dual-factor model of mental health.

**FIGURE 2:**
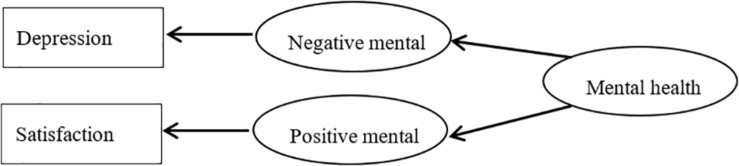
Satisfaction-depression of dual-factor model of mental health.

Based on DFM, we formulated a flourishing-depression DFM (Figure 1) and a Satisfaction With Life-depression DFM (Figure 2) that encompasses positive and negative mental health and features a “flourishing” and “Satisfaction With Life” index, pertaining to positive mental health, and a “depression” index, referring to negative mental health.

The SEM test results for Figure 1 were as follows: χ^2^/*df* = 3.043, goodness-of-fit index = 0.993, normed fit index = 0.996, comparative fit index = 0.997, incremental fit index = 0.997, adjusted goodness-of-fit index = 0.984, root mean square residual = 0.013, root mean square error of approximation = 0.031. The SEM test results for Figure 2 were as follows: χ^2^/*df* = 11.782, goodness-of-fit index = 0.979, normed fit index = 0.984, comparative fit index = 0.985, incremental fit index = 0.985, adjusted goodness-of-fit index = 0.955, root mean square residual = 0.055, root mean square error of approximation = 0.072. As required in SEM that when the number of samples is big enough, χ^2^/*df* value should be less than 5. The χ^2^/*df* value in Figure 1 is less than 5, whereas the value is as high as 11.782 in Figure 2. Parameters in Figure 1 are higher than those in Figure 2. These results demonstrate the structural validity of our flourishing–depression DFM.

### Dual-Dimensional Evaluation of College Students’ Mental Health

#### Four Categories of College Students’ Mental Health

Following Suldo and Shaffer (2008), these groups may be labeled (1) vulnerable, (2) troubled, (3) complete mental health—also called flourishing (Kelly et al., 2012), and (4) symptomatic but content—also labeled ambivalent (Eklund et al., 2011). Their categorization method was applied in our categorization of participants. And based on their PHQ-7 and FS scores, participants were divided into the following four groups: “flourishing” (high flourishing level without depression; *n* = 1,279, 61.9%), “vulnerable” (low flourishing level with depression; *n* = 365, 17.7%), “tolerance” (high flourishing with depression; *n* = 160, 7.8%), and “languish” (low flourishing with depression, *n* = 261, 12.6%) (Table 1).

**TABLE 1:** Proportion of college students’ mental health groups.

Depression	Flourishing level		
	Low	High	Total
Yes	365(17.7%)	1,279(61.9%)	1,644(79.6%)
No	261(12.6%)	160(7.8%)	421(20.4%)
Total	626(30.3%)	1,439(69.7%)	2,065(100%)

#### Six Categories of College Students’ Mental Health

According to Keyes (2007), six categories is a more specified categorization than four categories. Therefore, mental health statuses are divided into six categories according to participants’ flourishing levels (high, medium, and low) and depression symptoms (with or without symptoms). In our research, we further categorized FS scores as follows: ≥5 points represented a high flourishing level, 4–4.99 was a medium flourishing level, and <4 denoted a low flourishing level. The six groups were then categorized as follows: “flourishing” (high flourishing level without depression; *n* = 1,279, 61.9%), “mental illness and flourishing” (high flourishing level with depression; *n* = 160, 7.7%), “moderately mentally healthy” (medium flourishing level without depression; *n* = 311, 15.1%), “mental illness and moderately mentally healthy” (medium flourishing level with depression; *n* = 159, 7.7%), “languishing” (low flourishing level without depression; *n* = 54, 2.6%), and “mental illness and languishing” (low flourishing level with depression; *n* = 102, 7.5%) (Table 2).

**TABLE 2:** Six categories of college students’ mental health.

	Flourishing level			
Depression	Low	Medium	High	Total
No	54(2.6%)	311(15.1%)	1,279(61.9%)	1,644(79.6%)
Yes	102(4.9%)	159(7.7%)	160(7.7%)	421(20.4%)
Total	156(7.5%)	470(22.8%)	1,439(69.7%)	2,065(100%)

### Empirical Analysis of College Students’ Mental Health

Mental health evaluation measures such as the FS, SWLS, PIL-SF, MHC-SF, and PHQ-7 have all been applied in assessing college students’ positive and negative mental health statuses. Moreover, our research has demonstrated statistical significance using four distinct categorizations (*p* < 0.001). Further comparison between these two approaches shows that the scores from the FS, SWLS, MHC-SF, and PIL-SF are higher for the flourishing group (*n* = 1,279), the vulnerable group (*n* = 365), the symptomatic but content group (*n* = 160), and the languish group (*n* = 261). The differences in PHQ-7 scores pertaining to negative psychological indicators were also found to be statistically significant (*p* < 0.001) (Table 3).

**TABLE 3:** Comparison of college students’ positive and negative mental states across the four categories.

Mental health evaluation measure	Flourishing group	Vulnerable group	Tolerance group	Languishing group	*F*	*Partial eta squared*
FS-CV	6.04 ± 0.58	4.22 ± 0.62	5.63 ± 0.58	3.90 ± 0.79	1,439.85***	0.677
SWLS	5.53 ± 0.92	3.81 ± 0.81	4.77 ± 1.15	3.34 ± 1.01	609.54***	0.470
PIL-SF	5.52 ± 0.86	4.34 ± 0.74	5.02 ± 0.80	4.03 ± 0.82	356.75***	0.342
MHC-SF	4.08 ± 0.64	2.99 ± 0.76	3.65 ± 0.76	2.48 ± 0.79	529.99***	0.435
PHQ-7	0.51 ± 0.32	0.70 ± 0.29	1.50 ± 0.44	1.65 ± 0.49	1,018.74***	0.597

***p < 0.001.

*FS-CV, Flourishing Scale, Chinese version; MHC-SF, Mental Health Continuum, short form; PHQ-7, Patient Health Questionnaire, seven-item depression scale; PIL-SF, purpose in life test, short form; SWLS, satisfaction with life scale.*

Such scales as FS, SWLS, PIL-SF, MHC-SF, and PHQ-7 were also applied to evaluate participants’ mental health statuses using six distinct categorizations, the result of which demonstrated statistical significance (*p* < 0.001).

*Post hoc* analysis based on FS score reveals no significant difference in terms of high vulnerable and languishing group and general vulnerable and general tolerance group, but there were pairwise differences in other groups, with flourishing group **>** high tolerance group > general vulnerable and general tolerance group > high vulnerable and languishing group.

*Post hoc* analysis based on SWLS and PIL-SF scores demonstrates no significant difference between general vulnerable and general tolerance group, whereas significant difference can be found between all other groups, with flourishing group **>** high tolerance group > general vulnerable and general tolerance group > high vulnerable group > languishing group.

*Post hoc* analysis based on MHC-SF score shows significant difference among all groups, with flourishing group **>** high tolerance group > general vulnerable group > general tolerance group > high vulnerable group > languishing group.

*Post hoc* analysis based on PHQ-7 score showed no significant difference between high vulnerable and general vulnerable group, with flourishing group **<** high vulnerable and general vulnerable group < high tolerance group < general tolerance group < languishing group. Our analysis also reveals statistical significance with respect to the six categories (*p* < 0.001) (Table 4).

**TABLE 4:** Comparison of the college students’ positive and negative mental states between the six categories.

Mental health evaluation measure	Flourishing group	High vulnerable group	General vulnerable group	General tolerance group	High tolerance group	Languishing group	*F*	*Partial eta squared*
FS-CV	6.04 ± 0.58	3.14 ± 0.79	4.41 ± 0.33	4.36 ± 0.32	5.63 ± 0.58	3.17 ± 0.74	1198.80***	0.744
SWLS	5.53 ± 0.92	3.08 ± 0.97	3.93 ± 0.71	3.81 ± 0.73	4.77 ± 1.15	2.60 ± 0.94	423.208***	0.507
PIL-SF	5.52 ± 0.86	3.90 ± 0.80	4.41 ± 0.70	4.33 ± 0.70	5.02 ± 0.80	3.58 ± 0.78	235.449***	0.364
MHC-SF	4.08 ± 0.64	2.35 ± 0.92	3.10 ± 0.67	2.78 ± 0.66	3.65 ± 0.76	2.00 ± 0.73	367.095***	0.471
PHQ-7	3.60 ± 2.23	4.61 ± 2.62	4.95 ± 1.92	11.25 ± 3.21	10.53 ± 3.07	12.18 ± 3.73	615.471***	0.599

***p < 0.001.

## Discussion

This study proposed to verify a flourishing–depression combination DFM. The results showed that the selected indicators fit well with flourishing levels as a positive indicator and depression as a negative indicator. The results also demonstrated the structural validity of our flourishing–depression DFM, which was found to be able to reflect college students’ mental health status reliably. Additionally, college students’ mental health statuses were divided into four and six categories according to the DFM, and we found that college students experience great differences in life satisfaction, sense of life, positive psychology, flourishing levels, and depression. These results also further demonstrated the efficacy of the DFM.

Our study’s two proposed categorizations, through groupings of four and six, both revealed significant differences with respect to positive psychology (including flourishing levels, life satisfaction, and sense of life) and negative psychology (specifically, depression). Equally, though, results from our study were similar to those obtained by previous researchers. Eklund et al. (2011) found that mentally healthy college students scored high in positive psychological indicators (such as offering thanks, hopes), whereas morose college students typically manifested more negative behaviors (such as alcohol abuse). Our results further verified that our postulated categorization by four and six groupings based on the DFM provided distinction as well as effectiveness. In particular, categorization through reference to six groups offers a more specified and feasible mental health screening and prevention approach. For colleges and universities with limited resources, such categorization can help narrow the scope of screening and better identify at-risk individuals. In most other circumstances, our proposed four-category model could be adopted for use in mental health education and intervention.

According to the traditional PTH model, only those who have PTH symptoms require intervention. In our research, 20.4% of college students manifested symptoms of depression and would require intervention. According to the DFM, asymptomatic individuals are not necessarily high in positive psychological levels, and those who are high in such levels could still exhibit PTH symptoms. According to our four-category development of the DFM, both troubled (12.6%) and vulnerable (17.7%) groups would require intervention. Although they did not manifest PTH symptoms, they were low in flourishing levels and social psychological function and could therefore develop mental problems in the future. However, under traditional mental health assessments, these people are often ignored. In our research, 7.7% of college students had depression symptoms, but they were high in flourishing levels and could restore themselves quickly. Therefore, psychological intervention would not be necessary for them. Thus, compared with traditional mental health assessments, a DFM can discriminate mental illness and health more fully and, with particular reference to our research, categorize college students’ mental states more specifically. Using this approach, colleges and universities could undertake more effective prevention and intervention measures and thereby reduce the occurrence of psychological crisis events.

In previous studies, great differences have been found according to the application of different dual-factor scales. Percentages of mentally healthy college students have been found to be 23.8–61.4%; vulnerable students, 18.7–59.6%; symptomatic but content students, 4.8–12.4%; and troubled students, 4.4–21% (Peter et al., 2011; Renshaw and Cohen, 2014; Antaramian, 2015). In China, the PHQ-9, a Generalized Anxiety Disorder assessment (GAD-7), and a General Health Questionnaire (GHO-12) combination were used to assess negative indicators, and the Edinburgh Happiness Scale was used as a positive indicator, and it was discovered that the percentage of the population that could be categorized as mentally healthy was 69.8%; vulnerable was 7.3%; symptomatic but content was 13.5%; and troubled was 9.4% (Liu et al., 2017). Wang et al. (2016), in their investigation of college students’ mental health, used anxiety and depression in a general mental health scale as negative indicators and a sense of happiness and life satisfaction as positive indicators, and identified the percentage of the population who were mentally healthy to be 31.3%, with vulnerable at 28.2%, symptomatic but content at 14.7%, and troubled found to be 25.8%. In light of these studies’ varying results, we infer that, through differing screening criteria, a great deal of inconvenience can be introduced unnecessarily into mental health assessments and interventions. Consequently, we conclude that an appropriate, unified DFM scale is necessary for successful mental health screening.

In our sample, the percentage of mentally healthy individuals was found to be 61.9%, which is similar with results presented in Dong et al.’ (2014) and Liu et al.’ (2017) studies, but different from the findings of Hai et al. (2015) and Wang et al. (2016). Furthermore, based on our results, we would recommend exercising graded intervention for the troubled (12.6%) and vulnerable (17.7%) groups, making better use of the available mental health resources. If we needed to further distinguish the urgency of care required by individuals, we would refer to the results obtained using the six-category DFM and apply more focused intervention for the troubled (4.9%) and severely vulnerable (partly healthy group I, 2.6%) populations, rendering our mental health screening more operational and to the point. In addition, from a feasibility perspective, there are only 15 items in our flourishing–depression combination DFM, compared with more than 20 items in previous models. Our questionnaire is simpler and can be more easily used within different populations, and so could be used more widely.

However, as we only used cross-sectional methods, as regards the questionnaire and the model validation and longitudinal qualitative methods, such as follow-up observations, future clinical interviews are still required to ensure predictive validity and empirical validity. Additionally, more population groups need to be included in future studies to verify our model’s efficacy.

## Conclusion

Our flourishing–depression combination DFM demonstrates good structural fitness and applicability and can offer guidance regarding college students’ mental health screening and interventions. For institutions with limited resources, categorization by four can be utilized for mental health screening, and for institutions with enough resources, categorization by six can be utilized in screening for more detailed mental health statuses.

## Data Availability Statement

The raw data supporting the conclusions of this article will be made available by the authors, without undue reservation.

## Ethics Statement

The studies involving human participants were reviewed and approved by Academic Ethics Committee of Southern Medical University. The patients/participants were fully informed the nature of our research and participate in this study voluntarily.

## Author Contributions

RX and CZ contributed substantially to the conception and design of the study. RX and QL collected the data and designed the analysis. RX, CZ, and QL drafted the manuscript. RX and YH contributed to the revision of the manuscript. XZ designed the study and provided critical revision of the article. All authors contributed to the article and approved the submitted version.

## Conflict of Interest

The authors declare that the research was conducted in the absence of any commercial or financial relationships that could be construed as a potential conflict of interest.
